# Oral microbiota in youth with perinatally acquired HIV infection

**DOI:** 10.1186/s40168-018-0484-6

**Published:** 2018-05-31

**Authors:** Jacqueline R. Starr, Yanmei Huang, Kyu Ha Lee, C. M. Murphy, Anna-Barbara Moscicki, Caroline H. Shiboski, Mark I. Ryder, Tzy-Jyun Yao, Lina L. Faller, Russell B. Van Dyke, Bruce J. Paster, for the Pediatric HIV/AIDS Cohort Study

**Affiliations:** 1 Forsyth Institute, 245 First St, Cambridge, MA 02142 USA; 2 Department of Oral Health Policy and Epidemiology, Harvard School of Dental Medicine, Boston, MA USA; 3 Department of Oral Medicine, Infection, and Immunity, Harvard School of Dental Medicine, Boston, MA USA; 4 Department of Pediatrics, David Geffen School of Medicine, University of California Los Angeles, Los Angeles, CA USA; 5 Department of Orofacial Sciences, School of Dentistry, University of California San Francisco, San Francisco, CA USA; 6 Center for Biostatistics in AIDS Research, Harvard T.H. Chan School of Public Health, Boston, MA USA; 7 Ginkgo Bioworks, Boston, MA USA; 8 Tulane University School of Medicine, New Orleans, LA USA

**Keywords:** Perinatally infected HIV, Pediatric, Oral microbiome, *Corynebacterium*

## Abstract

**Background:**

Microbially mediated oral diseases can signal underlying HIV/AIDS progression in HIV-infected adults. The role of the oral microbiota in HIV-infected youth is not known. The Adolescent Master Protocol of the Pediatric HIV/AIDS Cohort Study is a longitudinal study of perinatally HIV-infected (PHIV) and HIV-exposed, uninfected (PHEU) youth. We compared oral microbiome levels and associations with caries or periodontitis in 154 PHIV and 100 PHEU youth.

**Results:**

Species richness and alpha diversity differed little between PHIV and PHEU youth. Group differences in average counts met the significance threshold for six taxa; two *Corynebacterium* species were lower in PHIV and met thresholds for noteworthiness. Several known periodontitis-associated organisms (*Prevotella nigrescens*, *Tannerella forsythia*, *Aggregatibacter actinomycetemcomitans*, and *Filifactor alocis*) exhibited expected associations with periodontitis in PHEU youth, associations not observed in PHIV youth. In both groups, odds of caries increased with counts of taxa in four genera, *Streptococcus*, *Scardovia*, *Bifidobacterium*, and *Lactobacillus*.

**Conclusions:**

The microbiomes of PHIV and PHEU youth were similar, although PHIV youth seemed to have fewer “health”-associated taxa such as *Corynebacterium* species. These results are consistent with the hypothesis that HIV infection, or its treatment, may contribute to oral dysbiosis.

**Electronic supplementary material:**

The online version of this article (10.1186/s40168-018-0484-6) contains supplementary material, which is available to authorized users.

## Background

Emerging research on the role of the gut microbiota in HIV infection highlights a complex and clinically important relationship. Microbes in the lower gastrointestinal tract and vagina have been associated with the acquisition of HIV [1]. By suppressing host immune function, HIV may cause microbial dysbiosis, which has been shown to influence HIV progression [1, 2].

Alteration in the oral microbiota is well known to have negative consequences including periodontitis, oral candidiasis, oral herpes lesions, and Kaposi’s sarcoma lesions [3, 4], all of which reduce quality of life and act as sentinel signs of underlying HIV/AIDS progression. Conversely, maintaining a healthy mucosal barrier, including through host-microbiota interactions, may help mitigate clinical symptoms of HIV disease [5–7].

Although analysis of oral salivary samples and lingual and subgingival plaque samples has shown overall similar microbial composition between HIV-positive and HIV-negative adults, some differences are notable [8–11]. For example, *Haemophilus parainfluenzae* was more prevalent and *Streptococcus mitis* less prevalent in HIV-positive adults than in HIV-negative adults [5, 10]. In the lingual microbiome, species of *Veillonella*, *Prevotella*, *Megasphaera*, and *Campylobacter* were associated with untreated HIV infection, which was also inversely associated with putative commensal species of *Streptococcus* and *Neisseria* [11]. Yet, collectively, little is known about the oral microbiome and its relationship to oral health sequelae in adults with HIV/AIDS, and even less is known about its relevance in HIV-infected youth. In a recent study of HIV-infected and HIV-uninfected children, there were few observed differences in phyla between the two groups; however, the small sample size (*n* = 16) limits firm conclusions [12].

One challenge to studying the relationship between perinatally acquired HIV infection and the oral microbiota is that children are born with HIV, before they have an established microbiome. In addition, perinatally HIV-infected (PHIV) children are repeatedly exposed to their HIV-infected mothers, whose microbiome appears to be altered compared with women uninfected with HIV [13–15]. In turn, the maternal microbiome influences the establishment of the microbiome of the children [16]. PHIV children are also placed on antiretroviral therapy (ART) early in life, with frequently shifting regimens. Although ART should help protect immune function, these medications may result in loss of protective bacteria and thereby allow the emergence of pathogenic species [5, 8, 17]. To capitalize on an appropriate comparison group that helps address these challenges, we conducted a sub-study within the Pediatric HIV/AIDS Cohort Study (PHACS), in which both HIV-infected and HIV-uninfected children were perinatally exposed to maternal HIV infection and, subsequently, to ART.

In a previous publication from the PHACS, we observed that caries but not periodontal disease was more common in PHIV youth compared with HIV-exposed but uninfected (PHEU) youth [18]. The present study focuses on the differences in oral microbiomes between the two groups. We address two primary questions: first, does oral microbial community composition differ in PHIV versus PHEU youth? Second, do caries-associated or periodontitis-associated organisms differ in PHIV versus PHEU youth?

## Methods

### Study design and population

The Oral Health Protocol was a cross-sectional study within the Adolescent Master Protocol of the Pediatric HIV/AIDS Cohort Study (PHACS; www.phacsstudy.org). The Adolescent Master Protocol is an ongoing prospective cohort study at 15 US clinical sites, designed to determine the health effects of HIV infection and ART on youth perinatally exposed to HIV. The AMP included a comparison group of perinatally HIV-exposed, uninfected (PHEU) youth. Details about the overall study and adolescent cohort have been published elsewhere [19, 20]. Briefly, age at enrollment for PHIV and PHEU was 7 to 15 years. Regularly scheduled visits included audio computer-assisted structured interviews for sexual behavior, physical examination, and chart reviews for medication, diagnoses, CD4 counts, and viral load. In this oral health sub-study, participants were enrolled from September 2012 through January 2014; ages ranged from 10 to 22 years at the time of biospecimen sampling [18, 21].

Examinations by dentists at each site were standardized and calibrated as previously described [21–23].

### Microbial sampling

Participants were instructed not to eat, smoke, floss, drink anything besides water, or brush their teeth for 90 min prior to sample collection. Subgingival plaque samples for each participant were then collected at the mesial buccal aspect of the first permanent upper left and lower right molars, if fully erupted, or from the first fully erupted tooth mesial to the first permanent molar site. The examiner collected subgingival plaque samples by first drying the sites with cotton, then placing a sterile endodontic paper point in the sulcus of the two sites for 10 s. The two samples were pooled into one cryovial, immediately put on ice, and within 4 h, frozen at − 80 °C for storage.

The oral microbiomes of parents and caretakers of the adolescent participants were not analyzed in this study.

### Caries and periodontal parameter end points

The vast majority of participants had no primary teeth; therefore, we combined information on permanent and primary dentition before categorizing the participants regarding the presence of caries (any versus none). Based on the periodontal parameters clinical attachment loss and probing depth, and using the CDC-AAP criteria, periodontitis was defined as present or absent [24].

### DNA isolation

Using sterile forceps, we removed paper points from the cryovials and placed them in 1.5-ml Eppendorf tubes that contained 200 μl of Tris buffer, pH 7.5. The blunt ends of the paper points (i.e., no bacteria) were secured by the caps, leaving the points immersed in the buffer. The vials were vortexed for 30 s. Cells were spun down at 14,000×*g* for 5 min. Pellets were suspended in 200 μl of fresh Tris. DNA was isolated from clinical samples by using a modified protocol of a DNA Purification Kit following instructions from the manufacturer (MasterPure, Epicentre Biotechnologies, Madison, WI, USA). Prior to steps that use the kit, the modified protocol uses Ready-Lyse™ Lysozyme Solution (Epicentre, cat. no. R1802M) for overnight incubation. Total DNA yields ranged from 200 to 500 ng per clinical sample. Technical replicates of DNA isolations were not performed.

### 16S rDNA sequencing

Universal primers (forward: 341F, reverse: 806R) targeting the V3 to V4 region of the 16S rRNA genes were used for PCR amplification of bacterial DNA. Fifty nanograms was used for each initial PCR reaction. Controls without added DNA were run as negative controls. AMPure beads were used for purification of amplicons. Libraries (100 ng of PCR product) were pooled, gel purified, and quantified by using quantitative PCR (qPCR). A modified protocol as described by Caporaso et al. [25] was used for 16S rDNA sequencing by using the Illumina platform on a MiSeq, resulting in 33,974,989 total initial reads that ranged from 13 to 339,805 per sample [26].

16S rDNA sequencing reads were first trimmed and filtered by using the built-in “fastqPairedFilter” function of DADA2 version 1.4 with the following parameters: truncLen = c(235,235), trimleft = 5, maxN = 0, maxEE = 0.75, truncQ = 2 [27]. The read pairs were then processed through the de-noising, pair-merging, and chimera-removing steps of the DADA2 pipeline by using default parameters. After dropping samples with < 200 reads, the total reads remaining were 18,448,552, ranging from 1330 to 230,039 per sample. A dataset including sequences in the GenBank database as of January 22, 2017, that matches with 99% identity and 99% coverage to the curated Human Oral Microbiome Database (HOMD; v14.51) reference rDNA sequences, was constructed [28]. This dataset was used as the training dataset for taxonomy classification up to genus level by using a naive Bayesian classifier [29] implemented in DADA2. Species-level classification was achieved via a string search for exact match to the above GenBank dataset.

We removed 16S rDNA sequences that, after this process, remained unmatched, an average of 0.1% of the total reads, and excluded 196 taxa with relative abundance < 10^−5^.

### Statistical analysis

Two hundred seventy-nine samples of either type were available for analysis. We excluded 25 (19 PHIV and 6 PHEU) participants from analysis due to antibiotic use within the prior 3 months, leaving 254 (154 PHIV and 100 PHEU) samples for analysis.

### Phylogenetic trees for the most abundant taxa

The relative abundance of each taxon for each sample was calculated as a simple proportion (including unmatched reads). We constructed phylogenetic trees for the 50 most abundant taxa plus taxa that illustrated differences noted below. Phylogenetic trees were generated using the Clustal V (weighted) method by using the Lasergene MegAlign program (DNASTAR, Madison WI). 16S rDNA sequences used to generate phylogenetic trees were obtained from HOMD [28].

### Microbial community diversity

Separately for PHIV and PHEU youth, we estimated three measures of taxonomic diversity by using QIIME [30]: rarefaction curves for samples with ≥ 65,000 sequences; the Simpson diversity index (1-D), which ranges from 0 to 1 and is higher when communities are more diverse; and the Shannon diversity index, which measures both richness and evenness and increases as there are more and more evenly distributed taxa. We performed *t* tests to compare differences in diversity between the PHIV and PHEU groups (Stata, version 12.1).

### Differences in microbial counts between PHIV and PHEU youth

For these analyses, we excluded 23 taxa present in fewer than 10 subjects in both groups combined, of which five taxa were not present in any PHEU participants (the reference group). For each taxon, we compared mean counts in PHIV and PHEU youth by fitting negative binomial regression models adjusted for age, sex, indicator of a dental visit in the previous year, race (white or other), ethnicity (Hispanic or not), and the total number of sequencing reads [31]. Regression results were reported as estimated rate ratios, the fold change in counts in the two groups. We set the significance level at 0.05 within each set of comparisons by applying a step-up Benjamini and Hochberg procedure [32]. Analyses were performed at both the species and genus levels.

Significance thresholds alone do not distinguish between true and false positive findings. To filter out likelier false positive findings, we applied two procedures to the set of “significant” results: the false positive report probability (FPRP) [33] and the Bayesian false discovery probability (BFDP) [34]. We used prior probabilities of 0.001, 0.01, and 0.05. On the result plots, we indicated as noteworthy taxa that met the significance threshold and also met any of the following criteria: (1) both the FPRP and the BFDP were < 0.5, (2) the FPRP was < 0.2, or (3) the BFDP was < 0.2. For these calculations, we used prior probabilities of 0.01 and, for the FPRP, expected rate ratio of 1.5.

### Differences in caries- or periodontitis-associated taxa between PHIV and PHEU youth

We tested whether the strength of association between odds of periodontitis (or caries) and each taxon was different in PHIV and PHEU youth. We fit logistic regression models with either periodontitis or caries as the dependent variable in relation to HIV status and levels of each taxon, in turn, in separate models. We log_10_ transformed the counts of each taxon, after replacing zeros with half the taxon’s minimum non-zero value. In addition to the covariates included in main analyses, models included the total counts for each sample and a multiplicative interaction term (HIV group times the count for that taxon). The *p* value for the test of interaction was compared to significance threshold of 0.05 [32]. We considered the primary analyses to be those focused on species putatively etiologic for either periodontitis (*n* = 9 species) or caries (*n* = 11 species). We also performed further exploratory analyses for all species.

## Results

### Phylogenetic diversity

**Fig. 1:**
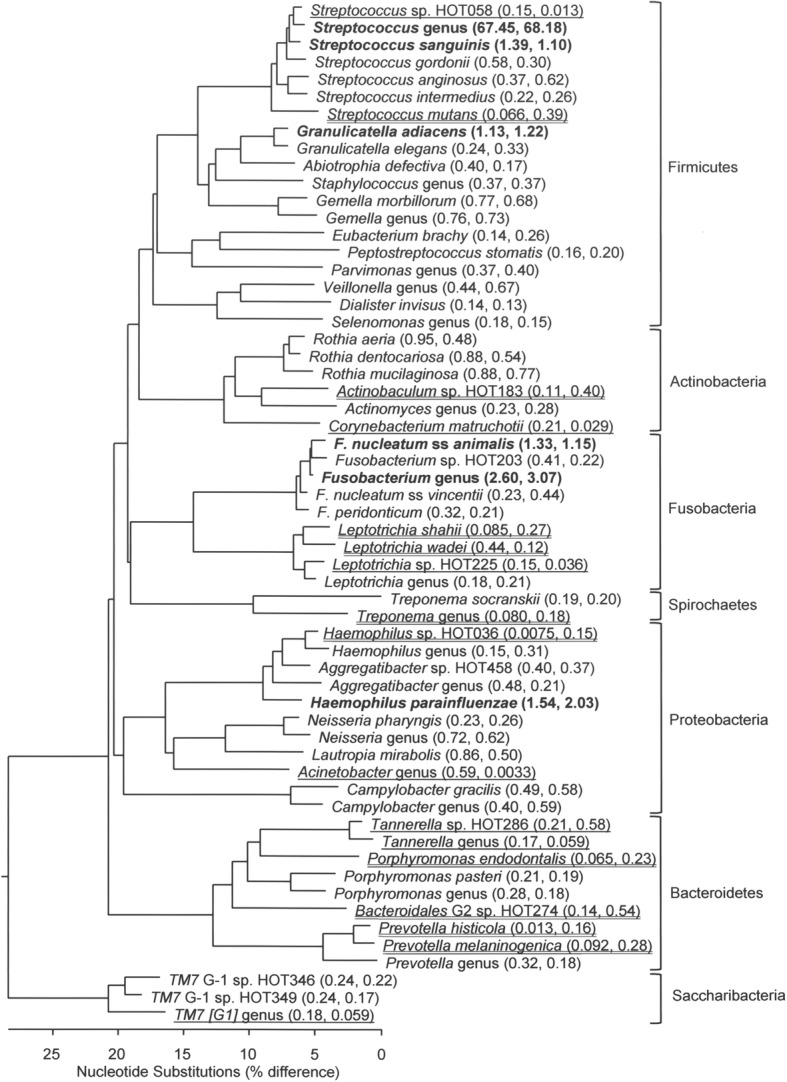
Phylogenetic tree depicting bacterial diversity of the most prevalent bacterial taxa in subgingival plaque samples of youth perinatally HIV-exposed and uninfected (PHEU) and perinatally HIV-infected (PHIV). Taxa are grouped into seven bacterial phyla indicated by brackets on the right. Predominant taxa found only in PHIV are noted by a single underline and those found only in PHEU are noted by a double underline. Numbers after taxa represent relative abundance (PHEU, PHIV). Taxa detected > 1% in relative abundance are noted in bold. Marker bar represents % difference in nucleotide sequence

Species of *Streptococcus*, including *S. sanguinis*, represented over 65% of the total taxa detected. Other abundant taxa included *Granulicatella adiacens*, species of *Fusobacterium*, including *Fusobacterium nucleatum* ssp. *animalis*, and *Haemophilus parainfluenzae* (Fig. 1).

### Richness and alpha diversity

**Fig. 2:**
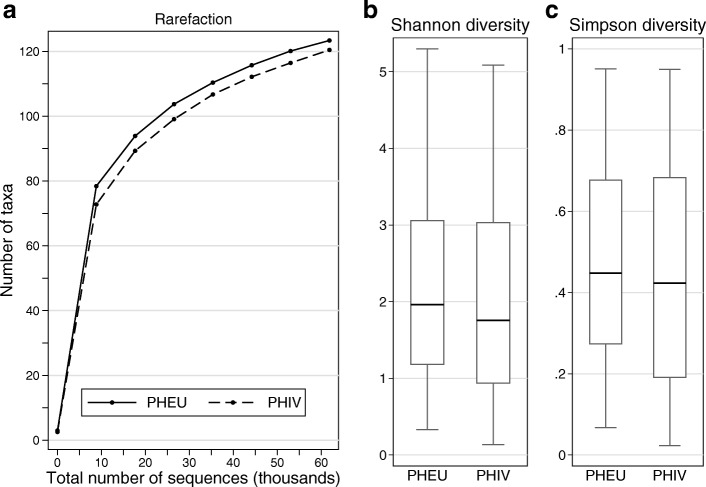
Three measures of microbial community diversity in subgingival plaque samples from youth perinatally exposed to HIV and uninfected (PHEU) and perinatally HIV-infected (PHIV). (**a**) Rarefaction curves show that richness (number of taxa detected versus number of sequences per sample) is similar for PHEU and PHIV. This analysis was restricted to subjects whose samples had ≥ 65,000 sequences (PHIV, *n* = 75; PHEU, *n* = 46). Alpha diversity for microbial taxa’s abundance based on the Shannon index (**b**) and Simpson index (**c**), by HIV infection status (PHIV, *n* = 154; PHEU, *n* = 100). Diversity for PHIV youth was comparable to, though very slightly lower than that for, PHEU youth (*p* > 0.3 for comparison of either index based on a *t* test)

Rarefaction curves were nearly identical for PHIV (*n* = 154) and PHEU (*n* = 100) (Fig. 2a). The Shannon and Simpson diversity indices also differed little between PHIV and PHEU, though samples from the PHIV group exhibited slightly lower diversity, on average (*p* > 0.3 for either index, Fig. 2b, c).

### Differences in microbial counts between PHIV and PHEU youth

**Fig. 3:**
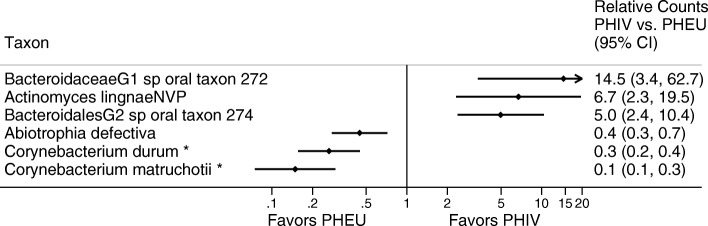
Ratios of average counts of microbial taxa detected in subgingival plaque samples from youth perinatally HIV-exposed and uninfected (PHEU) and perinatally HIV-infected (PHIV)

Adjusted group differences between PHEU and PHIV in average counts at the species level met the significance threshold for six taxa (Fig. 3 and Additional file 1: Table S1). PHIV-associated species included two Bacteroidetes species not yet cultivated (phylotypes), *Bacteroidaceae* G1 HOT 272 and Bacteroidales G2 HOT274, and *Actinomyces lingnae*. PHEU-associated species included two species of *Corynebacterium* (Actinobacteria), and *Abiotrophia defectiva* (Firmicutes). In genus-level analyses, in addition to the genera for the species already mentioned, three other genera also met the significance threshold, *Desulfobulbus*, *Mycoplasma*, and *TM7 G5* (Additional file 1: Table S2). At both the species and genus levels, only *Corynebacterium* met the noteworthiness threshold.

### Differences in periodontitis- or caries-associated organisms between PHIV and PHEU youth

Both the PHIV and PHEU groups had similar prevalence of periodontitis (~ 30%; Table 1). Several known periodontitis-associated taxa exhibited expected positive associations with periodontitis in PHEU, whereas these associations were not observed in PHIV, e.g., *P. nigrescens*, *T. forsythia*, *A. actinomycetemcomitans*, and *F. alocis* (Table 2)*.* For all periodontitis-associated organisms combined, for every tenfold increase in counts, the odds of periodontitis were increased 11% (95% CI 0, 24) among the PHEU youth, whereas no increase was observed in the PHIV group (*p* = 0.175 for test of PHIV-PHEU difference in odds ratios; Table 2).

**Table 1:** Prevalence of caries and periodontitis in youth perinatally HIV-infected (PHIV) or perinatally HIV-exposed, uninfected (PHEU) included in plaque sample analyses

	PHEU (*N* = 100)		PHIV (*N* = 154)	
Periodontitis	No caries	Any caries	No caries	Any caries
	*N* (%)	*N* (%)	*N* (%)	*N* (%)
No	38 (38.0)	32 (32.0)	34 (22.1)	68 (44.2)
Yes	17 (17.0)	13 (13.0)	24 (15.6)	28 (18.2)

**Table 2:** Microbial associations with periodontitis in perinatally HIV-infected (PHIV) vs perinatally HIV-exposed, uninfected (PHEU) youth in subgingival plaque samples

Species	PHEU OR (95% CI)^a^	PHIV OR (95% CI)^a^	Interaction *p* value
*Prevotella nigrescens*	2.1 (1.1, 4.0)	0.9 (0.6, 1.5)	0.06
*Tannerella forsythia*	2.0 (1.1, 3.4)	1.0 (0.7, 1.5)	0.06
*Desulfobulbus* genus	0.0 (0.0, 5.1)	0.9 (0.4, 1.9)	0.19
*Dialister invisus*	1.6 (1.0, 2.6)	1.2 (0.8, 1.7)	0.25
*Aggregatibacter actinomycetemcomitans*	1.1 (0.5, 2.4)	0.5 (0.1, 1.7)	0.26
*Filifactor alocis*	1.5 (0.8, 2.8)	1.0 (0.6, 1.5)	0.27
*Treponema denticola*	1.5 (0.9, 2.6)	1.1 (0.8, 1.7)	0.35
*Porphyromonas gingivalis*	0.7 (0.2, 3.3)	0.3 (0.1, 1.3)	0.42
*Streptococcus anginosus*	1.1 (0.8, 1.7)	1.2 (0.9, 1.7)	0.77
*Streptococcus sobrinus*	0.0 (Undefined)	0.0 (Undefined)	Undefined
All species combined	1.1 (1.0, 1.2)	1.0 (0.9, 1.1)	0.18

^a^*OR* odds ratio associated with each tenfold increase in counts, *CI* confidence interval

Sixty-two percent of PHIV youth had caries compared with 45% of PHEU youth (Table 1). The odds of having any caries was increased in both PHIV and PHEU youth as counts of taxa in four caries-associated genera increased, *Streptococcus*, *Scardovia*, *Bifidobacterium*, and *Lactobacillus* (Table 3). Across the *Veillonella* genus, species of *Veillonella* were inconsistently associated with odds of caries, and the OR estimates were also inconsistent between the PHIV and PHEU groups.

**Table 3:** Microbial associations with caries in perinatally HIV-infected (PHIV) vs perinatally HIV-exposed, uninfected (PHEU) youth in subgingival plaque samples

Species or genus	PHEU OR (95% CI)^a^	PHIV OR (95% CI)^a^	Interaction *p* value
*Veillonella* sp. oral taxon 780	0.7 (0.4, 1.4)	1.3 (0.8, 2.1)	0.16
*Veillonella parvula*	1.3 (0.6, 2.6)	0.7 (0.4, 1.3)	0.22
*Veillonella* genus^b^	0.8 (0.5, 1.3)	1.2 (0.8, 1.7)	0.28
*Veillonella rogosae*	0.6 (0.2, 1.2)	0.7 (0.3, 1.6)	0.61
*Lactobacillus* genus^b^	1.2 (0.4, 4.0)	1.8 (0.6, 5.3)	0.64
*Veillonella atypica*	1.2 (0.7, 2.1)	1.4 (0.8, 2.4)	0.67
*Lactobacillus salivarius*	2.3 (0.7, 7.9)	1.6 (0.6, 4.2)	0.68
*Streptococcus mutans*	1.4 (0.9, 2.3)	1.3 (0.9, 1.8)	0.70
*Bifidobacterium dentium*	1.2 (0.5, 3.0)	1.5 (0.7, 3.1)	0.76
*Scardovia wiggsiae*	1.2 (0.6, 2.2)	1.2 (0.7, 2.0)	0.92
*Streptococcus sobrinus*	–	1.1 (0.3, 3.7)	0.98
All taxa combined	1.0 (0.9, 1.2)	1.1 (1.0, 1.1)	0.79

^a^*OR* odds ratio associated with each tenfold increase in counts, *CI* confidence interval

^b^Taxa noted at the genus level include reads matched to the given genus that were not identified at the species level, i.e., subtracting out any sequences for that genus that were also identified at the species level

Neither for the primary analyses of nine periodontitis-associated taxa and 11 caries-associated taxa nor in exploratory analyses of all taxa did any individual species meet the significance threshold testing stratum-specific differences, i.e., whether estimates of association between odds of periodontitis (or caries) and levels of each taxon were different between PHIV and PHEU (Additional file 1: Tables S3 and S4).

## Discussion

The large cohort of youth perinatally infected with HIV allowed for a comprehensive investigation of the oral microbiome in these youth compared with a suitable control group of youth also perinatally HIV exposed but not infected. The bacterial taxa detected in both groups were, for the most part, similar, and many taxa were those that are typically detected in healthy oral sites. For example, subgingival plaque contained *Streptococcus anginosus*, *Streptococcus intermedius*, species of *Tannerella*, and species of *Treponema*, all known to be found in healthy individuals [35, 36].

We do not claim that PHEU participants have the same health status as non-HIV infected youth who were never exposed to HIV. Indeed, PHEU youth are appropriate controls precisely because they were exposed to their HIV-infected mothers at birth, similar to the PHIV group. PHEU, as well as PHIV, youth differ from non-HIV-exposed youth in reportedly having higher mortality [37], altered natural killer cell function [38], increased risk of infections [39], impaired vaccine responses [40], and lower CD4 counts [37]. Thus, the results should be unconfounded by factors underlying differences between HIV-exposed and HIV-unexposed children.

A strength of this study is that we sought to bolster the interpretation of results by applying other statistical filters in addition to significance testing. Although it may not be generally appreciated, the chance that a “significant” result is falsely positive increases with the nearness of the *p* value to the significance threshold and decreases with the statistical power and the strength of the scientific hypothesis being tested. These relationships can be formalized mathematically and used to estimate two types of false positive report probabilities, FPRP [33], and the BFDP [34].

Among the six species meeting the significance threshold, only two met false reporting thresholds, *Corynebacterium durum* and *Corynebacterium matruchotii*, for which average counts were 75–85% lower in PHIV versus PHEU youth. Counts of *Corynebacterium* identified only to the genus level exhibited a similar 87% decrease (reported in online supplement). *Corynebacterium* spp. have been observed consistently at higher abundance in orally healthy participants compared with those with oral disease [41, 42]. Commensal species of *Corynebacterium* have been shown to inhibit colonization and growth of oral pathogens such as *Streptococcus pneumoniae* by producing free fatty acids [43]. Further speculation as to a specific role for *Corynebacterium* arises from visualization of plaque communities via spectral-imaged fluorescent in situ hybridization (FISH). In healthy sites, one end of these filamentous microorganisms attaches to the tooth surface, appearing to anchor a multi-genus consortium organized around the *Corynebacterium* cell [44]. It is possible these differences noted in the relative abundance of *Corynebacterium* partially explain the higher prevalence and levels of caries observed in PHIV versus PHEU in the PHACS cohort [21]. The higher prevalence of caries in PHIV compared with PHEU group may seem at odds with the much lower relative abundance of caries-causing *Streptococcus mutans*, 0.39 versus 0.66 (Fig. 1), respectively. Like species of *Veillonella*, however, *C. matruchotii* can use lactic acid to modulate the local pH and may thus help to reduce the risk of caries induced by *S. mutans* and other acid-producing bacteria [45–47].

In previous studies in adult populations, other than in one study in which HIV infection and highly active antiretroviral therapy (HAART) were associated with alterations in the salivary microbiome of adults [8], few differences in oral microbiomes have been reported between subjects with and without HIV infection [9, 10]. Discrepancies may be due to subject population, including age, sample size, perinatal versus behavioral HIV infection, how microbiota were assessed, e.g., microarray analysis vs next-generation sequencing, or how data were analyzed.

It is tempting to speculate that in adolescents whose HIV infection is controlled by HAART or other therapies, the oral microflora would be similar to that of PHEU, and the microorganisms that cause oral disease would also be similar. Other than *Veillonella* spp*.*, known caries-associated microorganisms exhibited similar increases in odds of caries in PHIV and PHEU youth. Sensitivity might have been increased if we had sampled supra- rather than subgingival plaque, though the microbiomes of the two overlap greatly and stand apart from those of other oral sites in healthy subjects [48]. Known periodontal pathogens had much less consistent associations either within or across the groups, with levels of most exhibiting associations with odds of periodontitis only in PHEU and not in PHIV youth. Yet, it would be premature to conclude that therefore, etiologic factors must differ in PHEU and PHIV, in part because of the low prevalence and severity of periodontitis. Though periodontitis was present in ~ 30% of this PHACS subcohort, half of these participants had mild, half moderate, and none severe periodontitis based on CDC-AAP criteria [24]. Sampling at specific locations rather than comparing the microbiota at carious lesions and non-carious surfaces, or at the periodontium of healthy and diseased sites, likely decreased study sensitivity. And, testing differences in odds ratios between two groups (e.g., PHIV versus PHEU) typically requires larger sample sizes than testing whether one odds ratio is non-null. Thus, these results regarding associations with oral disease should be considered preliminary.

The children in PHACS comprise an important cohort for clarifying the history of HIV infection, oral microbial community composition, and their relation to common oral infectious disease. The data suggest there are fewer oral “health”-associated bacterial taxa in PHIV youth than in PHEU youth. The reduced abundance of *Corynebacterium* and *Abiotrophia* species may be why the PHIV group tended to have more caries. These results are consistent with the hypothesis that HIV infection, or its treatment, may contribute to oral dysbiosis.

## Conclusions

We compared subgingival plaque microbiota in youth with and without HIV infection. HIV-infected youth had fewer “health”-associated organisms such as *Corynebacterium* species; they did not exhibit expected associations of periodontitis with known periodontitis-associated organisms. HIV infection may promote oral dysbiosis.

## Additional file

Additional file 1: List of supplemental tables. **Table S1.** Ratio of average counts (at the species level) in perinatally HIV-exposed, infected (PHIV) versus perinatally exposed, uninfected (PHEU), with Bayesian False Discovery Probabilities and False Positive Report Probabilities. **Table S2.** Ratio of average counts (at the genus level) in perinatally HIV-exposed, infected (PHIV) versus perinatally exposed, uninfected (PHEU), with Bayesian False Discovery Probabilities and False Positive Report Probabilities. **Table S3.** Is the strength of association of periodontitis presence with oral taxa's counts the same inperinatally exposed, infected (PHIV) youth compared with perinatally exposed, uninfected (PHEU) youth?. **Table S4.** Is the strength of association of caries presence with oral taxa's counts the same in perinatally exposed, infected (PHIV) youth compared with perinatally exposed, uninfected (PHEU) youth?. (XLSX 214 kb)
